# Tramadol in drug-facilitated sexual assault: global case prevalence, pharmacology, and drug interactions

**DOI:** 10.1093/jat/bkag067

**Published:** 2026-07-21

**Authors:** David Stewart-Yates, Garth L Maker, John Coumbaros

**Affiliations:** School of Medical, Molecular & Forensic Sciences, Murdoch University, Murdoch, WA 6150, Australia; School of Medical, Molecular & Forensic Sciences, Murdoch University, Murdoch, WA 6150, Australia

## Abstract

Drug-facilitated sexual assault (DFSA) refers to sexual acts where the victim is incapacitated by the effects of psychotropic substances, causing impaired ability to prevent an attack and/or provide informed consent. To date, the level of understanding of the prevalence of the specific drugs involved in DFSA has been limited. This review examines global toxicological case data to assess the prevalence of tramadol, a widely prescribed opioid analgesic, in the context of DFSA. Tramadol was confirmed in reports across Africa, Oceania, Europe, the Middle East, and North America, with detection rates ranging from 1% to 33%. In these cases, 17 voluntary and involuntary drug combinations were identified including alcohol, opioids, benzodiazepines, Z-drugs, cannabinoids, and stimulants. These substances pose significant risk for both pharmacodynamic and pharmacokinetic interaction with tramadol, potentially altering plasma metabolite levels, clearance times and also central nervous system depression, facilitating DFSA and influencing forensic detection and interpretation of tramadol and its metabolites. Overall, this review underscores the significance of tramadol in DFSA casework and highlights the importance of drug–drug interactions in order to improve forensic analysis in DFSA casework and aid in legal action leading to victim support and closure.

## Introduction

The misuse of psychotropic substances to facilitate criminal acts, collectively termed drug-facilitated crime (DFC), poses significant global public health and safety concerns [1]. Commonly referred to as “date rape drugs,” “club drugs,” or “predatory drugs,” these psychotropic substances can impair a victim’s awareness, consciousness, judgement and memory, enabling the perpetration of crimes such as robbery, homicide, human trafficking, and most notably, drug-facilitated sexual assault (DFSA) [2, 3]. DFSA may involve the covert or forceful administration of incapacitating substances to facilitate sexual acts (proactive), exploitation of voluntary consumption (opportunistic) or a combination of both (mixed) [4]. Investigation of DFSA is commonly associated with delayed reporting, often up to several days or weeks following the event [5]. Additionally, appropriate measures to investigate drink spiking may not always be implemented, particularly in cases where sexual assault is not suspected [5]. In turn, the true prevalence of DFSA is difficult to determine and contribution of specific substances is limited.

Among the various substances commonly implicated in DFSA, tramadol has emerged as a substance with growing forensic significance [6]. Tramadol is a fully synthetic centrally acting opioid analgesic that is widely prescribed for moderate to severe pain [7]. Its central nervous system (CNS) depression effects, widespread availability and potential for misuse have led to its emergence in DFSA reports across the globe [8, 9]. Tramadol has a dual mechanism of action, acting on opioid receptors and blocking the reuptake of serotonin and norepinephrine to produce analgesia and sedation [10]. Tramadol is extensively metabolized in the liver by cytochrome P450 enzymes including CYP2D6, CYP3A4, and CYP2B6. Notably, CYP2D6 is responsible for the conversion of parent tramadol to its main active metabolite (*O*-desmethyltramadol) which has significantly more pharmacological activity. Tramadol can be combined with other CNS depressants such as alcohol or benzodiazepines, to potentiate central depression causing dizziness, sleepiness, unconsciousness, and impaired concentration, thinking, and judgement [11]. These effects may affect a victim’s ability to resist a DFSA attack or provide informed consent and therefore increases tramadol relevance in DFSA investigations.

In a DFSA investigation, analytical toxicology, and interpretive pharmacology serve to detect, identify, quantify, and interpret drugs within the context of the offence. Analytical toxicology is typically performed using sensitive hyphenated chromatography methods such as gas chromatography-mass spectrometry (GC-MS) and liquid chromatography-mass spectrometry (LC-MS) (including tandem mass spectrometry [LC-MS/MS] and high resolution quadrupole time of flight mass spectrometry [LC-QTOF-MS]), whereby biological and non-biological (e.g. alcoholic beverages) matrices are analyzed for the presence of DFSA-related substances and their metabolites [12–14]. Blood samples of 5–10 ml are preferred in cases where the victim has reported the incident within 2–48 h and may provide information into pharmacological effect, while urine (30–50 ml) can extend detection window up to 4 days following intake and is therefore a useful matrix for cases in which the victim has delayed reporting [15]. Beyond this time/detection window, hair (150–200 hairs at 1–3 cm segments) can be analyzed to demonstrate drug intake at least 4 weeks following the alleged assault [15, 16]. It must be noted, detection windows in biological material vary by drug and administered dose [13].

Accurate interpretation of toxicological data remains one of the most challenging tasks in forensic toxicology. Interpretation relies on pharmacodynamic and pharmacokinetic principles to infer drug exposure and to answer legal questions, however the impact of inter-individual variation in drug response and disposition, time delays in sample collections and other factors such as developed drug tolerance leads to considerable uncertainty in casework [9]. Failure to consider the impact of variation according to age, sex, ethnicity, body weight, disease status, genetic factors of drug metabolizing enzymes or transporters and drug combinations can result in inaccurate case interpretation, impacting justice outcomes and victim support.

Importantly DFSA cases typically involve a combination a drugs, increasing risk for drug–drug interaction (DDI). These interactions may influence the pharmacological effect (pharmacody­namic DDI) or absorption, distribution, metabolism, or elimination (phar­macokinetic DDI) of a victim drug [17]. A common site of metabolic DDI is competition for binding to CYP2D6, an enzyme which ­significantly impacts the pharmacokinetics of tramadol. Further, CYP2D6 is highly polymorphic, raising risks for drug–drug–gene interaction (DDGI), in which magnitude of DDI is further influenced by enzyme phenotype. Overall, drug interactions can influence tramadol metabolism and drug response, and the analyst’s ability to accurately interpret the case if the influence of inter-individual variation or environmental factors is not considered.

To date, several reviews have addressed the drugs involved, global prevalence, and analytical challenges associated with DFSA [3, 14, 18]. However, a significant gap in the knowledge base of tramadol exists. Therefore, the aim of this study was to overview the pharmacological profile of tramadol, evaluate its global prevalence in DFSA and identify key drug interactions relevant to tramadol in DFSA casework. Reported herein, this work establishes a more detailed and critical evaluation of tramadol’s role in DFSA, which is currently absent in the scientific literature.

## Methodology

PubMed and Scopus databases were searched using the terms “Drug Facilitated Sexual Assault,” “Drug Facilitated Crime,” “Chemical Submission,” “Drink Spiking,” “Opioid,” and “Tramadol” and combinations of these terms, with no publication date limits. Once an initial list had been produced, the reference lists were scanned to identify further relevant papers. Inclusion criteria included being published in English, with publications excluded if they were not in English, did not confirm tramadol in toxicological analysis or did not include DFSA, DFC or chemical submission in the report.

## Tramadol pharmacology

Tramadol is chemically identified as (1*RS,* 2*RS*)-2-[(dimethylamino)methyl]-1-(3methoxyphenyl)-cyclohexanol hydrochloride. It has a dual mechanism of action including μ-opioid receptor (MOR) agonism and monoaminergic modulation, specifically involving the inhibition of norepinephrine (NE) and serotonin (5-hydroxytryptamine [5-HT]) reuptake [19, 20]. Compared to other opioids, tramadol has a relatively weak affinity for MOR—approximately 10-fold less than codeine and 6000-fold less than morphine [21]. Its affinity for κ-OR (KOR) and δ-OR (DOR) is even weaker: 20-and 27-fold less, respectively [19]. Tramadol contains two chiral centers and is marketed as a racemic 1:1 mixture of the *R, R*-enantiomer ([+]-tramadol) and *S, S*-enantiomer ([-]-tramadol). The enantiomers of tramadol clinically contribute to its pharmacological effects, as (+)-tramadol exhibits higher affinity for MOR and preferentially inhibits 5-HT reuptake [22], while (-)-tramadol shows lower affinity for MOR and inhibits NE reuptake [23].

Agonism at MOR causes an increase in potassium conductance and a decrease in calcium conductance, interrupting the release of neurotransmitters such as glutamate and substance P between primary and secondary neurons, thereby preventing pain signal transmission [24, 25]. The non-opioid mechanism of tramadol has also been found to contribute to analgesia, as elevated synaptic NE levels inhibit nociceptive impulse transmission through α_2_-adrenergic receptors [26]. The metabolism of tramadol is significant for its pharmacological effect, undergoing P450-mediated metabolism into two active metabolites: *O*-desmethyltramadol (M1, ODT) and *N*, *O*-didesmethyltramadol (M5, NODT). These metabolites exhibit significantly higher MOR affinity compared to parent tramadol, with (+)-ODT displaying 700-fold and M5 displaying 24-fold higher affinity. In summary, parent tramadol has weak MOR agonism with serotonergic and noradrenergic activity, whereas its active metabolites demonstrate stronger MOR agonism but lack monoaminergic activity.

Tramadol can be administered by oral, intravenous or rectal routes and is available in both immediate-release (IR) and sustained-release (SR) formulations. In Australia, oral formulations consist of 50 mg IR and 50–300 mg SR preparations, with a recommended maximum daily dose of 400 mg. After oral administration, tramadol is rapidly absorbed in the intestine, achieving a bioavailability of ∼68% [10]. In healthy volunteers, a single 50 mg oral dose resulted in a maximum blood concentration (*C*_max_) of 156 ng/g at 2.2 h, while a 100 mg dose produced a *C*_max_ of 335 ng/g at 2.6 h [27]. ODT concentrations peaked slightly later, reaching *C*_max_ of 44 and 68 ng/g within 2.8 and 3.2 h, following administration of 50 and 100 mg tramadol, respectively [27]. The volume of distribution (*V_d_*) of tramadol is 2.6 L/kg in males and 2.9 L/kg in females, with a pattern of distribution to the brain, liver, kidneys, and lungs [28].

Bastami *et al.* assessed the final detectable time points of tramadol and ODT in blood [27]. Unchanged tramadol was detected up to 14 h after 50 mg and 21 h after 100 mg doses, while ODT was detectable for 10 and 19 h, respectively [27]. However, these values represent mean results, and inter-individual variability was substantial. For example, while the average final detectable point of unchanged tramadol following intake of 50 mg tramadol was 14 h, in some volunteers this time was either much shorter at 10 h or longer at 25 h. Similarly, *C*_max_ of 50 mg tramadol ranged from 1.5 to 3 h.

Tramadol undergoes extensive Phase I and II metabolic reactions, yielding at least 26 metabolites [29]. Phase I metabolism primarily catalyzed by P450 enzymes leads to at least 14 metabolites (M1–M11 and M31–M33), while Phase II reactions result in at least 12 metabolites, of which seven are conjugates with glucuronic acid (M12–M18) and five are conjugates with sulfonates (M19–M23). The Phase I metabolites: ODT, *N*-desmethyltramadol (M2, NDT), *N, N*-didesmethyltramadol (M3, NNDT), *N, N, O*-tridesmethyltramadol (M4, NNODT), and NODT represent the major metabolites detected in human urine following oral dose. Around 30% is eliminated as unchanged tramadol, while ODT and NDT represent the most abundant metabolites in urine [29].

An important factor in tramadol pharmacology is the involvement of CYP2D6, a highly polymorphic isoform. This isoform is responsible for the conversion of parent tramadol to its main active metabolite, ODT, and therefore allelic variations leading to altered enzyme activity can lead to significant variations in individual drug response and clearance. Individuals are classified into poor (PMs), intermediate (IMs), extensive (EMs), or ultra-rapid metabolizers (UMs). Poor metabolizers for CYP2D6 tend to produce 75% less (+)-ODT compared to normal metabolizers and hence, less analgesia [30, 31]. PMs have been observed in 5%–10% of Caucasian, 0%–19% of African and 0.2% of Asian populations [32, 33]. In contrast, UMs experience higher levels of (+)-ODT in blood and enhanced side effects [30] with a prevalence of 16% in Ethiopians, 20% in Saudia Arabians, 0.8%–3.6% in North Europeans, 4.3% and 4.5% in American Caucasians and African Americans, respectively, and 7%–10% in Mediterranean Europeans [33].

## Tramadol prevalence in DFSA

This review has identified 19 studies reporting the presence of tramadol within the context of chemical submission and drink spiking. This includes 14 countries within Africa, Oceania, Europe, the Middle East, and North America. Single studies were retrieved from Australia, Belgium, New Zealand, Norway, Northern Ireland, Italy, Poland, South Africa, Spain, Sweden, and the United States of America (USA), with two from Iran and three each from Denmark and France. Key data including crime type (DFSA, DFC, drink spiking), alcohol consumption and detection, opioid and tramadol prevalence, and drug combinations are summarized in Table 1. As DFSA is an example of DFC, and both are closely associated with drink spiking, data reporting tramadol under any of these three examples is included in this review. Further, the term chemical submission was used interchangeably with DFSA. The majority of victims in these cases were female with DFSA being the most reported crime type. Concentrations of tramadol and/or its metabolites were reported in only three (16%) of the included studies [5, 16, 34]. Consequently, meaningful comparison of concentrations between cases was not possible, and the present review focused primarily on prevalence, detection, and reported drug combinations. Additionally, limited data on limit of detection (LOD) was reported, with only four studies reporting LOD range from 0.0001 to 0.1 μg/ml in urine [34–37] and 0.02 μg/ml in blood [37].

**Table 1:** Global prevalence of tramadol in drug-facilitated crime, drink spiking, and drug-facilitated sexual assaults.

Country	Crime type(s)	Sample data	Toxicological specimen	Alcohol	Opioid prevalence	Tramadol prevalence	Tramadol drug combinations expected/determined	Ref.
Australia (2002–2003)	DFSA	76 suspected cases, 15 (19.7%) confirmed cases, 95% of victims were female	Blood/urine	77% reported alcohol consumption (37% confirmed by toxicology)	27% (Tramadol, oxycodone, codeine)	25% of opioid cases 7% of total cases	Diazepam, ethanol, and codeine (expected); cannabis and methamphetamine (unexpected)	[8]
Belgium (2017–2018)	DFSA	79 confirmed cases, 92% of victims were female, 5% male, and 3% gender-neutral	Blood/urine	46% reported alcohol consumption (19% confirmed by toxicology)	4% (Morphine, codeine, hydrocodone, tramadol)	33% of opioid cases 1% of total cases	Aspirin	[9]
Denmark (2009–2016)	DFC; DFSA	130 suspected cases, 25 (19.2%) confirmed cases, 76% of victims were female	Hair	NR	24% (Codeine, morphine, oxycodone, tramadol)	17% of opioid cases 4% of total cases	Codeine, morphine, zolpidem, lidocaine	[41]
Denmark (2015–2022)	DFSA	268 suspected cases, 231 (86%) confirmed cases, 98% of victims were female and 2% were male	Blood/urine/hair	95% reported alcohol consumption (45% confirmed by toxicology)	10% (Tramadol, codeine, morphine, fentanyl, oxycodone, methadone, buprenorphine, ketobemidone, heroin)	4% of total cases	NR	[43]
Denmark (2021–2022)	DFSA	38 suspected cases, 26 (68%) confirmed cases	Urine	NR	16% (Morphine, codeine, hydromorphone, methadone, buprenorphine, tramadol, heroin)	NR	NR	[42]
France (2001–2019)	DFC; DFSA; food/drink spiking	6497 suspected cases, 934 (14%) confirmed, 66% of victims were female and 34% were male	Blood/urine/hair/chemical vector	47% reported alcohol consumption (4% confirmed by toxicology)	6% (Codeine, buprenorphine, tramadol, pholcodine, morphine, methadone, dextromethorphan, propoxyphene, oxycodone, trimebutine)	17% of opioid cases 1% of total cases	NR	[45]
France (2003–2007)	DFC; DFSA; food/drink spiking	309 suspected cases, 158 (51%) confirmed cases, 56% of victims were female	Blood/urine/hair/chemical vector	NR	5% (Morphine, propoxyphene, pholcodine, tramadol)	13% of opioid cases 1% of total cases	NR	[44]
France (2021–2022)	DFC; DFSA; food/drink spiking	100 suspected cases, 32 (32%) confirmed cases	Blood/urine	90% reported alcohol consumption (54% confirmed by toxicology)	22% (Tramadol, codeine, methadone, buprenorphine)	50% of opioid cases 9% of total cases	Ethanol, buprenorphine, tetrahydrocannabinol, ketamine, MDMA, MDA, cetirizine, hydroxyzine, cocaine	[46]
Iran (2009–2010)	DFC; DFSA; food/drink spiking	53 confirmed cases, 85% of victims were male and 15% were female	Urine	NR	43% (Dextromethorphan, tramadol, methadone, codeine, levorphanol, morphine)	22% of opioid cases 9% of total cases	NR	[52]
Iran (2018–2019)	DFC; DFSA; food/drink spiking	40 confirmed cases, 70% of victims were male and 30% were female	Blood/urine	NR	NR (Morphine, methadone, tramadol)	20% of opioid cases 3% of total cases	NR	[53]
Italy (2018–2020)	DFSA	207 suspected cases, 92% of victims were female, 8% were male	Blood/urine	27% confirmed by toxicology	6% (Methadone and tramadol)	33% of opioid cases 2% of total cases	NR	[47]
New Zealand (2015–2018)	DFSA	162 suspected cases, 76 (47%) confirmed cases, 98% were female, 2% were male	Blood/urine	47% confirmed by toxicology	NR (Codeine, tramadol, morphine)	NR (detection in two blood and six urine samples)	NR	[40]
Northern Ireland (1999–2005)	DFSA	294 suspected cases, 90 (31%) confirmed cases	Blood/urine	51% confirmed by toxicology	27% (Codeine, morphine, dihydrocodeine, tramadol)	4% of opioid cases 1% of total cases	Codeine, trazodone	[48]
Norway (2018–2019)	Drink spiking	100 suspected cases, 15 (15%) confirmed, 62% of victims were female	Blood/urine	91% reported alcohol consumption (66% confirmed by toxicology)	7% (Tramadol)	100% of opioid cases 7% of total cases	NR	[5]
Poland (NR)	DFC; DFSA	12 suspected cases, 3 (25%) confirmed	Urine	NR	33% (Tramadol)	100% of opioid cases 33% of total cases	NR	[51]
South Africa (2013–2016)	DFSA	107 suspected cases, 72 (67) confirmed, 97% of victims were female	Blood/urine/hair	79% reported alcohol consumption (54% confirmed by toxicology)	5% (Tramadol, morphine, codeine, oxycodone)	40% of opioid cases 2% of total cases	Citalopram, venlafaxine	[39]
Spain (2016–2020)	DFSA	95 suspected cases, 95% of victims were female	Blood/urine/hair/clothing	55% confirmed by toxicology	4% (Morphine, tramadol)	NR	NR	[49]
Sweden (2003–2010)	DFSA	3212 suspected cases, 100% of victims were female	Blood/urine	25% confirmed by toxicology	1% (Buprenorphine, propoxyphene, ethyl morphine, codeine, methadone, tramadol, oxycodone)	NR	NR	[50]
USA (2015–2016)	DFSA	1000 suspected cases, 784 (78%) confirmed, 92% of victims were female	Blood/urine	31% confirmed by toxicology	13% (Morphine, hydromorphone, hydrocodone, oxycodone, methadone, fentanyl, codeine, buprenorphine, tramadol, heroin, oxymorphone, dihydrocodeine)	14% of opioid cases 2% of total cases	NR	[54]

Data are presented in alphabetical order by country.

### Africa

Tiemensma and Davies analyzed blood, urine or hair of 107 suspected DFSA incidents by LC-MS/MS in South Africa between 2013 and 2016 [38]. There were two tramadol cases, and in one case it was found in combination with other serotonin reuptake inhibitors, citalopram, and venlafaxine. Notably, majority of the incidents occurred in a private setting, including the assailant’s home (47%), friend’s home (11%), the victim’s own home (9%), car (7%), or hotel room (7%), involving up to 11 perpetrators.

### Oceania

In Australia, 76 cases of suspected DFSA were reported between 2002 and 2003 [8]. Toxicological analysis by GC-MS confirmed 15 (19.7%) cases with opioids, including tramadol, oxycodone, and codeine representing 27% of confirmed cases. Most victims were female (95%) and 82% involved drink spiking. Reported combinations of substances with tramadol included diazepam, ethanol and codeine, while cannabis and methamphetamine were detected but not reported as voluntarily ingested. In New Zealand, 76 (47%) of the 162 suspected cases of DFSA between 2015 and 2018 were confirmed to have unexpected toxicology in urine and blood samples [39]. Codeine was positive in five blood samples and 10 urine samples, tramadol was positive in two blood samples and six urine samples, while morphine was positive in one blood sample and two urine samples using LC-time of flight mass spectrometry (LC-TOFMS).

### Europe

Majority of the studies (68%) in this review were conducted in Europe, including Belgium, Denmark, France, Italy, Northern Ireland, Norway, Poland, Spain, and Sweden. In Belgium between 2017 and 2018, 79 confirmed DFSA cases were reported [9]. In these cases, morphine, codeine, tramadol, and hydrocodone were detected with opioid prevalence at 4%, carried out by ultra-high pressure LC-MS/MS (UPLC-MS/MS). Conversely, opioids were a common drug implicated in DFSA in Denmark, producing case frequencies from 10% to 24% between 2009 and 2022 [16, 35, 36]. In one study covering the years 2009–2016 conducted by Wang *et al.* 130 cases were suspected and 25 were confirmed by toxicology of hair samples [16]. Opioids, including codeine, morphine, oxycodone, and tramadol were confirmed by UPLC-MS/MS in 24% of the cases, with tramadol representing 17% of opioid cases. A complainant (female, age 30–50 years) presented with tramadol and *O*-desmethyltramadol hair concentrations of 0.045 and 0.008 ng/mg, respectively. In this case, tramadol was also combined with codeine (0.031 ng/mg), morphine, zolpidem (0.050 ng/mg), and lidocaine (0.024 ng/mg), which were found at hidden at the complainant’s residence and drugging was confirmed in court. Skov *et al.* report that between 2015–2022, 231 of the 268 suspected DFSA cases were confirmed by UPLC-MS/MS, with opioids implicated in 10% of cases [35]. In both reports, tramadol represented 4% of total cases. Unfortunately, in a report between 2021 and 2022, the exact frequency of tramadol could not be determined; however, opioids accounted for 16% of cases [36].

Djezzar *et al.* collected DFC data in France from 2003 to 2007 and 2001 to 2019, reporting similar opioid prevalence around 5% and 6%, respectively [40, 41]. In both reports, toxicological analysis were performed by validated LC-MS, LC-MS/MS, or GC-MS approaches. Tramadol was confirmed in one case between 2003 and 2007 and in nine cases between 2001 and 2019. Importantly, these studies also incorporated toxicological analysis of chemical vectors in 56 cases, which included alcoholic and non-alcoholic beverages, food, and cigarettes. The range of aggressions being facilitated from most to least frequent were sexual assault, theft, simple sedation, homicide, physical assault, kidnapping, psychological manipulation, and inheritance misappropriation.

More recently, Liautard *et al.* analyzed 100 suspected DFC cases admitted to Hotel-Dieu Hospital in Paris, between 2021 and 2022, with 70% suspecting sexual violence and the remainder suspecting robbery/theft [42]. Blood and urine samples were screened using LC-MS/MS, in which 32 of the suspected victims were confirmed victims of DFC, DFSA, drink spiking or a combination. Notably, opioid prevalence was much higher in this report at 22% compared to 5%. Tramadol was the most common opioid detected, accounting for 50% of opioid cases and 9% of total cases. Tramadol was found in combination with a range of other substances including CNS depressants such as ethanol, buprenorphine, tetrahydrocannabinol (THC), ketamine, hydroxyzine (and metabolite, cetirizine), as well as stimulants including cocaine, 3,4-methylenedioxyamphetamine (MDA), and 3,4-methylenedioxymethamphetamine (MDMA).

Following the investigation of 207 suspected DFSA victims between 2018 and 2020 in Italy, 94 victim samples were positive for alcohol, drugs or combination [43]. In this report, methadone and tramadol were the only opioids detected by LC-MS/MS. In Northern Ireland, toxicological analysis revealed an opioid case frequency of 27% between 1999 and 2005, with tramadol representing 4% of these cases [44]. In this study, tramadol was found in combination with codeine and trazodone, a serotonin reuptake inhibitor. In Norway, between 2018 and 2019, tramadol was the only opioid found in drink spiking cases and was detected with blood concentration by UPLC-MS/MS [5]. A 29-year-old female complainant presented to Oslo Accident and Emergency Outpatient Clinic, feeling tired, vomiting, disoriented, nauseous, and faint. The complainant reported not experiencing the anticipated pharmacological effects and therefore suspected drink spiking. In this case, alcohol was reported to be ingested but not detected and it was understood the complainant presented. Concentration of 211 nM of tramadol was found in blood sample; however, no urine sample was taken.

In Spain between 2016 and 2020, there was an opioid prevalence of 4%; however, tramadol’s individual prevalence was not reported [45]. In Sweden, between 2003 and 2010, there were 3212 suspected cases, with ethanol or other drugs being confirmed in 2194 cases [46]. Most victims presented with blood-ethanol concentration of 1.2 g/L, but a maximum concentration of 4.3 g/L was observed. Opioids were found in 1% of cases, however individual opioid data was grouped together so tramadol prevalence could not be determined. Toxicology was carried out by LC-MS. There were 12 cases of suspected DFSA in Poland, three cases were confirmed and tramadol was present in one urine sample at 4.51 µg/ml using GC-electron ionization MS (GC-EIMS) [34].

### Middle East

Currently, two case reports are available from Iran [47, 48]. This region was the only to report males to be the most frequent victims and theft to be the most reported DFC, followed by DFSA. Between 2009 and 2010, opioids were confirmed in 43% of DFC, DFSA, drink spiking or combination cases, with tramadol contributing 22% of opioid cases and 9% of total cases [47]. Other opioids detected were dextromethorphan, methadone, codeine, levorphanol, and morphine. Additionally, between 2018 and 2019, Moghaddam and Rahimi found tramadol to be implicated in 3% of cases [48]. In both reports, high performance-LC (HPLC) were the primary analytical method for toxicological assessment.

### North America

In the United States, Fiorentin and Logan documented 784 cases of DFSA with opioids being implicated in 13% of cases between 2015 and 2016 [37]. Tramadol was confirmed in 18 victims, with a number of other opioids also detected including morphine, hydromorphone, hydrocodone, oxycodone, methadone, fentanyl, codeine, buprenorphine, heroin, oxymorphone, and dihydrocodeine. In this report both blood and urine samples were screened by LC-QTOF-MS.

## Drug combinations and DDIs

In DFSA and drink spiking casework, multiple substances are commonly detected, arising from the beverages, spiked substances, and voluntarily consumed drugs (i.e. prescription or recreational drug[s]). Notably, tramadol interacts with over 760 drugs, raising concerns for pharmacodynamic and pharmacokinetic interactions [10]. Perhaps the most critical interactions involve induction or inhibition of the P450 enzymes [17]. Enzyme induction is a slow process which requires multiple doses and results in accelerated metabolism, while inhibition can occur from single dose exposure and involves a decrease in metabolism due to the inhibiting compound binding to active or allosteric site. Therefore, in DFSA casework inhibition metabolic DDIs are expected to be more common. CYP2D6, 3A4, and 2B6 are collectively responsible for metabolism of 70%–80% of marketed drugs, leading tramadol to be susceptible to DDIs when co-administered with inducers, inhibitors or substrates of these three enzymes. All three of these P450’s have identified inhibitors, while CYP2B6 and CYP3A4 also have known inducers [49]. Given tramadol is a partial pro-drug, interactions that affect the level of active metabolite produced are of significant clinical and forensic interest.

This review has identified a total of 17 drug combinations with tramadol. A subset of 10 has been chosen for further discussion based on relevance as a DFSA drug or substance of abuse, these include CNS depressants—ethanol, codeine, buprenorphine, morphine, diazepam, zolpidem, cannabis—as well as stimulants—cocaine, methamphetamine, and MDMA.

### Ethanol (alcohol)

Ethanol is the most commonly implicated substance in DFSA worldwide [8]. The reported levels of alcohol consumption per nation range between 46% and 95%; however, toxicological confirmation reveals a lower range of 4%–66%, likely attributed to the rapid metabolism of ethanol. Most suspected DFSA victims present with blood alcohol ranging between 0.06 and 0.4 g/100 ml at time of analysis [8, 39, 43, 44, 46]. Effects of ethanol vary with blood alcohol concentration (BAC): at 0.03–0.12 g/100 ml, mild euphoria, increased confidence and reduced inhibition occur; at 0.09–0.25 g/100 ml memory and judgement are impaired; 0.18 g/100 ml, confusion and memory loss occur; and above 0.25 g/100 ml, consciousness may be impaired [50].

As a CNS depressant, ethanol can potentiate central depression when combined with tramadol, enhancing pharmacodynamic effects [51]. Ethanol may also interact with opioid receptors and the serotonergic system, similar to tramadol, contributing pharmacodynamic interaction. The effect of ethanol on the release of opioids from controlled release formulations has gained particular attention. Traynor *et al.* investigated the impact of ethanol on the release of three controlled-release once-daily tramadol formulations: 200 mg Tridural^™^ tramadol HCl extended-release tablets, 200 mg Ultram® ER tablets and T-long® 200 mg capsules [52]. The release of tramadol from Ultram® tablets and T-long® capsules significantly increased by almost 40% and 75% and 215% and 115% after 4 h exposure to 20% and 40% ethanol, respectively. Conversely, release of tramadol from Tridural^™^ tablets decreased by around 30% and 20% in the presence of 20% and 40% ethanol. This difference is expected to be due to solubility variances in inactive ingredients in the formulations. Additionally, opioids, including tramadol, reduce gastrointestinal motility which can directly influence the absorption of drugs from GIT. Animal (rat) studies have shown that low doses of opioids led to increased ethanol consumption [53] and decreased absorption [54], attributable to decreased GIT emptying.

Although tramadol and ethanol do not share metabolic pathway, ethanol affects P450 enzymes, though the extent remains unclear. While its ability to induce CYP2E1 is well established and is linked to alcohol tolerance [50, 55], its effects on other enzymes, such as CYP3A4, are less characterized. Dic-Ijiewere and Osadolor [56] analyzed CYP3A4 activity in males (*n* = 82) with a history of alcohol (beer, spirit, wine, and gin) and tramadol concomitant use, finding the primary reasons for consumption were euphoric (77.4%) or aphrodisiac (58.1%) effects. Frequent drinkers were associated with significantly reduced serum CYP3A4 activity which would influence *N*-demethylation in tramadol metabolism. Similarly, Huang *et al.* found that ethanol inhibited CYP3A4 when co-administered with alprazolam, with molecular docking revealing a hydrogen bond formed between ethanol and SER119 residue, likely preventing substrate binding [57]. In contrast, Feierman *et al.* found evidence that ethanol can induce CYP3A4 in vitro (HepG2 cells) and in vivo (rats), which could significantly accelerate tramadol clearance [58].

Comparative studies between ethanol consumers and abstainers found that hepatic CYP3A4 was not affected as evidenced by clearance and half-lives; however, the weakly reduced oral bioavailability following administration of midazolam suggests potential intestinal CYP3A4 induction [55]. Gazzaz *et al.* reported no effect of 0.7 g/L of ethanol on hepatic CYP3A4 activity, but observed inhibition of intestinal CYP3A4 and hepatic CYP2D6 [59]. In this study, exposure to the opioid dextromethorphan was doubled due to CYP2D6 inhibition, which was most prevalent in EMs.

In summary, ethanol has been observed to inhibit and induce intestinal CYP3A4 and may inhibit, induce or produce no reaction in hepatic CYP3A4. A variance in the activity of CYP3A4 or CYP2D6 can significantly influence the metabolite profile of tramadol as well as its pharmacological effects. It is also important to note that effects are likely to be drug and dose-dependent, meaning research in this area should be a priority.

### Opioids

The opioids buprenorphine, codeine, and morphine were reported in combination with tramadol. Notably, codeine was identified in combination with tramadol in three cases—one each in Australia, Denmark and Northern Ireland. All three opioids are prescribed for moderate to severe pain, though buprenorphine may also be used to treat opioid use disorder owing to its slow onset of action, partial MOR agonism and longevity in the body [60]. Codeine is a weak MOR agonist that requires CYP2D6 bioactivation to form morphine, similar to tramadol. Morphine is a strong MOR with weak KOR and DOR agonist activity, whereas buprenorphine is a partial MOR agonist and KOR antagonist. The half-lives of these opioids significantly vary, with buprenorphine presenting a half-life of 38 h [61], whereas codeine and morphine range between 1–4 and 1–7 h, respectively [28].

Approximately 7% of codeine is converted to morphine (by CYP2D6), while 6%–8% is converted to norcodeine by CYP3A4 and 60%–70% is converted to codeine-6-glucoronide by UGT2B7 [62]. Around 90% of morphine is metabolized by glucuronidation by UGT2B7, to form morphine-3-glucoronide and morphine-6-glucoronide, but a minor pathway includes CYP3A4 conversion to the metabolite, normorphine [63]. Buprenorphine undergoes CYP3A4-mediated *N*-dealkylation to form norbuprenorphine, with both former compounds undergoing glucuronidation (UGT1A1) to give rise to buprenorphine glucuronide and norbuprenorphine glucuronide, respectively [64].

As buprenorphine, codeine and morphine all act on opioid receptors, there is potential for additive or synergistic effect when combined with tramadol, enhancing the opioid effects including sedation, euphoria, drowsiness, and increasing chance of respiratory depression and overdose. Given the mutual enzymatic pathways between the four opioids, there is potential for pharmacokinetic interaction in terms of competition for binding sites and potential inhibition at CYP3A4 and CYP2D6. For example, *O*-demethylation of tramadol to ODT was inhibited following co-administration with the opioid methadone, another CYP2D6 substrate [65]. This was revealed by lower ODT levels in the urine of volunteers, all of whom expressed EM CYP2D6 gene. Inhibition of tramadol metabolism can decrease its analgesic and sedative effects, but will increase the levels of parent tramadol, which is associated with adverse reactions including serotonin toxicity.

### Benzodiazepines and Z-drugs

The benzodiazepine diazepam and the Z-drug zolpidem were detected in combination with tramadol. Diazepam undergoes bioconversion by CYP3A4 and CYP2C19 [66] while zolpidem is mostly metabolized by CYP3A4, with CYP1A2 and CYP2D6 playing minor roles [67]. While both pharmacodynamic and pharmacokinetic interactions are likely to occur, the former has potential to play a more important role in opioid-benzodiazepine combinations [66]. Notably, both tramadol and diazepam have active metabolites. The potential pharmacodynamic interaction of diazepam and zolpidem on tramadol is enhanced central depression leading to increased sedation, respiratory depression, psychomotor impairment and adverse reaction [68–72]. Lagard *et al.* found the interaction between diazepam and tramadol to be mostly pharmacodynamic, but also found metabolism to be influenced, leading to prolonged exposure to tramadol and its metabolites [73]. This can directly influence the duration of the opioid effect and influence the window for detection.

### Cannabinoids

Cannabis was detected in combination with tramadol in cases from Australia and France. A clinical trial conducted by Bindler *et al.* found that combination of oral hydrocodone/acetaminophen (7.5/325 mg) and smoked cannabis, resulted in a more rapid absorption rate and a short elimination half-life of 137 min compared to 346 min [74]. Additionally, a pharmacodynamic interaction occurred, leading pain scores to be lower with opioid and cannabis combination. Additional in vitro cell microsomes expressing P450s and UGTs have identified that delta-9-THC, 11-carboxy-THC THC-COOH, 11-carboxy-THC-glucuronide (THC-COO-Gluc), cannabinol (CBD), and cannabidiol (CBN) inhibited a range of enzymes including CYP2D6, CYP3A4, CYP2B6, and UGT2B7 [75–78]. Inhibition of these P450s will delay clearance of tramadol, decreasing onset of analgesia by active metabolites and increase exposure to the drug.

### Stimulants

The sympathomimetic stimulants, cocaine, methamphetamine, MDA, and MDMA were also found in combination with tramadol. Although central depressants are more commonly encountered in DFSA casework, stimulants may lower inhibitions, increase sexual motivation and increase susceptibility to suggestion [79]. Therefore, they continue to emerge in DFSA casework. Amphetamines are extensively metabolized by P450-mediated pathways. Notably, CYP2D6 facilitates the conversion of meth­am­phet­amine to the active metabolites, 4-hydroxymethamphetamine and amphetamine [80]. MDMA is *O*-demethylated by CYP2D6, CYP1A2, and CYP3A4 to 4-dihydroxymethamphetamine (HHMA). HHMA is further converted to 4-hydroxy-3-methoxymethamphetamine (HMMA) and *N*-demethylation by CYP3A4 forms MDA [81]. Therefore, combination of these stimulants with tramadol presents the potential for pharmacokinetic interaction at CYP2D6 and CYP3A4, as well as increasing the risk of serotonin syndrome owing to serotonergic activity.

Faria *et al.* found that combination of tramadol and MDMA resulted in no significant effect on serotonin levels, but a significant decrease in cell viability [82]. In animal (rat) model it was found that concomitant administration of tramadol and MDMA did not influence the elimination rate of tramadol when given intravenously or intraperitoneally [83]. However, the authors also found that, while intravenous tramadol led to metabolic inhibition, causing less conversion to M1, M2, and M5 and producing higher tramadol levels, when given intraperitoneally the absorption of tramadol reduced and the duration of detectable active metabolites (ODT and M5) was significantly increased. In addition, in a rat hepatocyte model *O*-desmethyltramadol levels were significantly reduced when combined with MDMA, and given *N*-desmethyltramadol levels were increased, potential induction of CYP3A4 could not be ruled out [84].

## Conclusion

Tramadol is an opioid analgesic that may be used to incapacitate victims and facilitate crime, yet published research concerning its prevalence in DFSA and metabolic interactions when combined with other drugs expected within this context is limited. This review provides critical insight into the global prevalence of tramadol in crime facilitation, revealing case detection rates as high as 33% and identifies 17 drug combinations with tramadol implicated in DFSA. Interpretation of tramadol within DFSA experiences several challenges including delayed reporting, limited availability of quantitative analytical data, frequency of concomitant drug intake, and impact of inter-individual variation in pharmacodynamics and pharmacokinetic process between victims. Given tramadol’s widespread prescription and availability, its true involvement in DFSA is likely underestimated, and the extent of drug combinations under realized. Expanding scientific efforts to report toxicology findings in regions not covered by this review, such as Asia and South America, is essential to gain a more comprehensive understanding of the role of tramadol in DFSA and further identify the key pharmacokinetic and pharmacodynamic interactions that can influence case detection and interpretation. This review has highlighted the potential for other drugs to influence the pharmacology of tramadol, modifying active metabolite levels and influencing clearance times. While all combinations present an interaction risk, ethanol, cannabis, and codeine present the highest in terms of potential influence on pharmacological processes, ability to detect and interpret the results and the frequency in which they have been confirmed. Important limitations of this review include the absence of quantitative analytical data and case specific drug combinations in the majority of DFSA reports. As drug interactions are drug and dose dependent, further research should study DDIs that may affect tramadol metabolism, to improve forensic analysis in DFSA casework and aid in legal action and victim support. Overall, the complex pharmacological profile of tramadol, its widespread availability and its susceptibility to drug interactions make it a significant concern in DFSA investigations and further research is required to enhance public safety, prevention measures and detection techniques.

## Data Availability

There are no new data associated with this article.
